# DNILMF-LDA: Prediction of lncRNA-Disease Associations by Dual-Network Integrated Logistic Matrix Factorization and Bayesian Optimization

**DOI:** 10.3390/genes10080608

**Published:** 2019-08-12

**Authors:** Yan Li, Junyi Li, Naizheng Bian

**Affiliations:** College of Computer Science and Electronic Engineering, Hunan University, Changsha 410082, China

**Keywords:** dual-network integrated logistic matrix factorization, Bayesian optimization, lncRNA and disease associations

## Abstract

Identifying associations between lncRNAs and diseases can help understand disease-related lncRNAs and facilitate disease diagnosis and treatment. The dual-network integrated logistic matrix factorization (DNILMF) model has been used for drug–target interaction prediction, and good results have been achieved. We firstly applied DNILMF to lncRNA–disease association prediction (DNILMF-LDA). We combined different similarity kernel matrices of lncRNAs and diseases by using nonlinear fusion to extract the most important information in fused matrices. Then, lncRNA–disease association networks and similarity networks were built simultaneously. Finally, the Gaussian process mutual information (GP-MI) algorithm of Bayesian optimization was adopted to optimize the model parameters. The 10-fold cross-validation result showed that the area under receiving operating characteristic (ROC) curve (AUC) value of DNILMF-LDA was 0.9202, and the area under precision-recall (PR) curve (AUPR) was 0.5610. Compared with LRLSLDA, SIMCLDA, BiwalkLDA, and TPGLDA, the AUC value of our method increased by 38.81%, 13.07%, 8.35%, and 6.75%, respectively. The AUPR value of our method increased by 52.66%, 40.05%, 37.01%, and 44.25%. These results indicate that DNILMF-LDA is an effective method for predicting the associations between lncRNAs and diseases.

## 1. Introduction

Long non-coding RNAs (lncRNAs) are a class of non-coding RNAs (ncRNAs) that are more than 200 nucleotides (nt) in length and do not encode proteins [1]. lncRNAs were originally thought to be genomic transcriptional noise without biological function [2]. Later, more and more evidence indicated that lncRNAs play an important role in many key biological processes, such as translation and post-translational regulation, cell differentiation, proliferation and apoptosis, and epigenetic regulation [3]. Meanwhile, mutations and dysregulation of lncRNAs can cause a variety of human diseases [4,5], including diabetes [6], AIDS [7], and many types of cancer, such as hepatocellular carcinoma [8], lung cancer [9], prostate cancer [10], breast cancer [11], and bladder cancer [12]. Therefore, predicting the potential associations between lncRNAs and diseases helps to explore the complex pathogenesis and etiology of disease at the molecular level and effectively improves the quality of disease diagnosis, treatment, and prevention.

In recent years, several lncRNAs function–disease relationship databases have been established. lncRNAdb [13], lncRNADisease [14], lnc2Cancer [15], and NONCODE [16] are some examples. However, the known lncRNA–disease relationship is still rare, and the use of biological experiments to explore lncRNA–disease associations is both time-consuming and expensive. Using computational methods to infer the potential associations between lncRNAs and diseases has become an effective prior method for biological experiments.

Recently, many computational models have been proposed to predict potential lncRNA–disease associations, which can roughly be divided into three categories. The first class of methods is based on machine learning to predict potential associations. Chen et al. [17] proposed LRSLDA, a semi-supervised learning method based on Laplacian regular least squares. This method does not require a negative sample. However, the problem of parameter selection for combining two classifiers has not been well solved. LDAP [18] uses a support vector machine classifier to predict potential lncRNA–disease associations based on lncRNA similarity and disease similarity. Yu et al. [19] constructed a global quadruple network and a global tripartite network by integrating various biological information. Based on these two global networks, the novel probability model NBCLDAbased on the naive Bayesian classifier was proposed.

The second category is based on biological network models. Heterogeneous data have become a hot topic in recent years. These models tend to construct heterogeneous networks using disease-associated genes/miRNAs or predict new associations between lncRNAs and diseases using multi-data source information fusion. Liang et al. [20] proposed a new method, TPGLDA, for predicting lncRNA–disease associations using a lncRNA–disease–gene tripartite map. It integrates gene–disease associations and lncRNA–disease associations and can effectively identify potential lncRNA–disease associations. Chen et al. [21] proposed an improved restart random walk model IRWRLDA, which integrates multiple data sources including lncRNA expression similarity, functional similarity, Gaussian interaction profile kernel similarity, and disease semantic similarity to predict lncRNA–disease associations. Gu et al. [22] established a global network random walk model GrwLDA, which predicts potential lncRNA–disease associations by integrating disease semantic similarity, lncRNA functional similarity, and known lncRNA–disease associations. These data fusion-based methods have achieved significant improvements over methods that use a single data source.

The third category is some methods based on matrix completion. MFLDA [23] decomposes data matrices of heterogeneous data sources into low-rank matrices via matrix tri-factorization to explore and exploit their intrinsic and shared structure. However, it cannot predict lncRNAs that are not associated with any disease or diseases that are not associated with any lncRNA. SIMCLDA [24] models the lncRNA–disease associations’ prediction problem as a recommended task and uses the induction matrix completion method to solve it.

The lncRNA–disease association matrix and the drug–target association matrix are generally sparse matrices with less known associations. The sparsity of the lncRNA–disease dataset used in this paper is 97.36%, which was obtained from 1-540/(115*178) (115 lncRNAs, 178 diseases, and 540 known associations, sparsity = 1-540/(115*178)), and the sparsity of the four benchmark datasets is 99.01%, 96.55%, 97.00%, and 93.59%, respectively, in drug–target interaction prediction [25]. With regard to the sparse characteristics of the drug–target matrix, neighborhood regularized logistic matrix factorization (NRLMF) was adopted in [26] to predict drug–target interactions, and the effect was significant. NRLMF has also been successfully applied to the prediction of the associations between miRNA–disease [27] and lncRNA–protein [28,29]. Based on NRLMF, dual-network integrated logistic matrix factorization (DNILMF) introduced a drug similarity network and target similarity network to improve the accuracy of prediction [26]. However, the DNILMF prediction effect was greatly affected by the parameter setting. The method of setting parameters based on experience had significant limits in [26]. Because the Gaussian process mutual information algorithm (GP-MI) [30], an advanced Bayesian optimization method, has been successfully applied to the parameter optimization of the logistic matrix factorization model and brings about positive results [31], this paper adopts the GP-MI algorithm to optimize the parameters for DNILMF.

**Figure 1:**
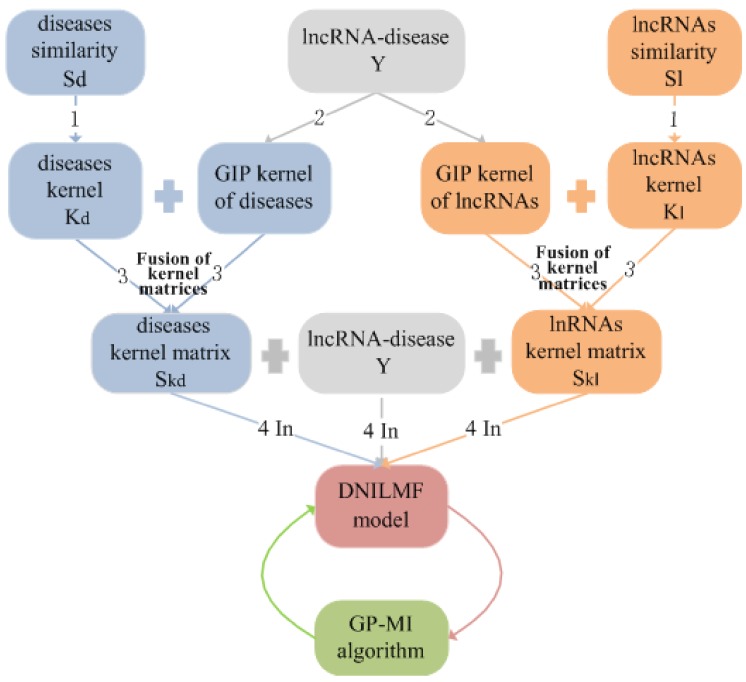
The flowchart of dual-network integrated logistic matrix factorization-lncRNA–disease association (DNILMF-LDA). **Step 1**: converting the calculated lncRNAs’ similarity matrix and the diseases’ similarity matrix to the corresponding kernel matrix; **Step 2**: calculating the Gaussian interaction profile kernel matrix of lncRNAs and diseases, respectively; **Step 3**: fusing two kernel matrices corresponding to the lncRNAs and the diseases respectively into one kernel matrix; **Step 4**: constructing the DNILMF model with the lncRNA–disease associations matrix, lncRNAs, and diseases kernel matrices as the input data. In order to ensure the optimal performance of the algorithm, the Gaussian process mutual information (GP-MI) algorithm is used to select parameters. GIP, Gaussian interaction profile.

The advantages of using DNILMF-LDA to predict lncRNA–disease associations are: (1) logistic matrix factorization, especially suitable for binary variables and sparsity problems, is used to model the interaction probability of each lncRNA–disease pair; (2) two different similarity kernel matrices of lncRNAs and diseases are fused into a composite kernel matrix by nonlinear fusion technology, and then, the fused kernel matrices are integrated into the model; (3) the lncRNAs’ and diseases’ similarity networks are introduced in the model; the flowchart of DNILMF-LDA given in Figure 1.

## 2. Materials

### 2.1. lncRNA–Disease Associations Matrix

The original lncRNA–disease association dataset was downloaded from the lncRNADisease [14] database, which integrated 687 experimentally-validated lncRNA–disease associations between 246 diseases and 369 lncRNAs. The diseases without disease ontology (http://disease-ontology.org/) and lncRNAs without expression profiles in ArrayExpress [32] (http://www.ebi.ac.uk/arrayexpress/) were filtered out, and 540 experimentally-validated lncRNA–disease associations between 115 lncRNAs and 178 diseases were obtained. The lncRNA–disease association matrix is represented by *Y*.

### 2.2. lncRNA Expression Similarity Matrix and Disease Semantic Similarity Matrix

More than 60,000 expression profiles from 16 human tissues were downloaded from ArrayExpress [32]. The Spearman correlation coefficient between any two lncRNAs in 115 lncRNAs was calculated and was used as the expression similarity for this pair of lncRNAs [17]. The expression similarity matrix of all lncRNAs is represented by $S_{l}$.

The semantic similarity of diseases is often used to predict potential lncRNA–disease associations. The semantic similarity of the disease in this paper was calculated with the method in paper [33]. Each disease was represented by a directed acyclic graph (DAG) containing all relevant annotated items, which came from the National Library of Medicine (http://www.nlm.nih.gov/mesh). The semantic similarity of two diseases is based on both the addresses of these diseases in DAG graphs and their semantic relations with their ancestor diseases. The DOSE package provided us with the method to calculate semantic similarities among diseases [34]. The semantic similarity matrix of the disease is represented by $S_{d}$.

### 2.3. Similarity Kernel Matrices

Kernel matrices of lncRNAs and diseases were constructed for nonlinear kernel fusion. The construction of kernel matrices consisted of two steps.

The first step was to convert the lncRNAs’ and diseases’ similarity matrices into kernel matrices. In this step, $S_{l}$ and $S_{d}$ were converted to kernel matrices by:

(1) converting $S_{l}$ and $S_{d}$ to the corresponding symmetric matrices, $S_{sym}=\left(S+S^{T}\right)/2$;

(2) transforming the symmetric matrices obtained in the first step into semi-positive definite matrices by adding multiple small identity matrices [35]. The transformed lncRNAs’ and diseases’ kernel matrices are represented by $K_{l}$ and $K_{d}$, respectively.

The second step was to calculate the Gaussian interaction profile (GIP) kernel matrix of lncRNAs and diseases. $Y_{li}$ and $Y_{lj}$ represent the interaction profile of lncRNA iand lncRNA j, which are the ith row and jth vector of association matrix *Y*. The distance between these two vectors was computed as their GIP kernel. In this step, for a given lncRNA–disease associations matrix *Y*, the GIP kernel $K_{gip}^{l}$ between lncRNAs was calculated according to Formula (1) [35]:

(1) $$K_{gip}\left(l_{i},l_{j}\right)=\exp\left(-\frac{{∥Y_{li}-Y_{lj}∥}^{2}}{\sigma }\right)$$

where ∥·∥ represents the Euclidean distance and σ represents the kernel bandwidth of the Gaussian spectrum. In our work, the value of σ was set to one. GIP kernel $K_{gip}^{d}$ between diseases was calculated using the same method.

### 2.4. Fusion of Similarity Kernel Matrices

The purpose of similarity kernel matrices’ fusion is to merge $K_{gip}^{l}$ and $K_{l}$ into a kernel matrix and merge $K_{gip}^{d}$ and $K_{d}$ into another kernel matrix. The steps of kernel fusion [36,37] are:

(1) Normalize and symmetrize the above four kernel matrices. Taking the fusion steps between $K_{gip}^{l}$ and $K_{l}$ as an example, the resulting matrices are denoted by $P^{\left(1\right)}$ and $P^{\left(2\right)}$.

(2) Construct local similarity matrix $L^{\left(1\right)}$ and $L^{\left(2\right)}$ of $K_{gip}^{l}$ and $K_{l}$ by Formula (2):

(2) $$L^{\left(1\right)}\left(i,j\right)=\left\{\begin{array}{c} \frac{P^{\left(1\right)}\left(i,j\right)}{\sum_{k\in N_{i}}P^{\left(1\right)}\left(i,k\right)},j\in N_{i}\\ 0,\;\;\mathrm{others}\;\; \end{array}\right.$$

where $P^{\left(1\right)}\left(i,j\right)$ represents the ith row and jth column element in matrix $P^{\left(1\right)}$. $N_{i}$ denotes the nearest neighbors of the current target *i*. The number of nearest neighbors was set to 3 according to experience. The similarity between lncRNAi and non-nearest neighbors was zero. Finally, $L^{\left(1\right)}$ and $L^{\left(2\right)}$ can be obtained;

(3) Update $P^{\left(1\right)}$ and $P^{\left(2\right)}$ iteratively by Formulas (3) and (4). Iteration step *t* was set to two by experience.

(3) $$P_{t}^{\left(1\right)}=L^{\left(1\right)}P_{t-1}^{\left(2\right)}{\left(L^{\left(1\right)}\right)}^{T}$$

(4) $$P_{t}^{\left(2\right)}=L^{\left(2\right)}P_{t-1}^{\left(1\right)}{\left(L^{\left(2\right)}\right)}^{T}$$

(4) After the iterations, $P_{t}^{\left(1\right)}$ and $P_{t}^{\left(2\right)}$ were averaged and normalized as the final kernel matrix of diseases, denoted as $S_{d}^{k}$. $S_{l}^{k}$ was calculated using the same method.

## 3. Methods

### 3.1. Problem Formalization

In this paper, the collection of lncRNAs is represented by $L={\left\{l_{i}\right\}}_{1}^{m}$, and the collection of diseases is represented by $D={\left\{d_{j}\right\}}_{1}^{n}$, where *m* and *n* are the number of lncRNAs and diseases, respectively. The associations between lncRNAs and diseases are represented by a binary matrix $Y\in R^{m\times n}$. When lncRNA $l_{i}$ was experimentally verified to be associated with disease $d_{j}$, $y_{ij}=1$, otherwise, $y_{ij}=0$. $L^{+}=\left\{l_{i}|\sum {}_{j=1}^{n}y_{ij}>0,\forall 1\leq i\leq m\right\}$ is the collection of positive lncRNAs, and $D^{+}=\left\{d_{i}|\sum {}_{i=1}^{m}y_{ij}>0,\forall 1\leq j\leq n\right\}$ is the collection of positive diseases. Thus, $L^{-}=L/L^{+}$ is the collection of lncRNAs with no known association with all diseases. $D^{-}=D/D^{+}$ is the collection of diseases with no known association with all lncRNAs. $S_{l}^{k}\in R^{m\times m}$ is the final similarity kernel matrix of lncRNAs, and $S_{d}^{k}\in R^{n\times n}$ is the final similarity kernel matrix of diseases. The purpose of this paper is to predict lncRNA–disease interaction probabilities and rank candidate lncRNA–disease pairs based on predicted probabilities. The higher ranked lncRNA–disease pairs are most likely to be correlated.

### 3.2. Prediction of lncRNA–Disease Associations Using the DNILMF Model

The lncRNAs’ kernel matrix $S_{l}^{k}$, diseases’ kernel matrix $S_{d}^{k}$, and lncRNA–disease association matrix *Y* are the input data for the DNILMF model to infer potential lncRNA–disease associations. lncRNAs and diseases were mapped to the r-dimensional shared potential space, where r < min(m, n). Latent vectors $u_{i}\in R^{1\times r}$ and $v_{j}\in R^{1\times r}$ represent the characteristics of lncRNA $l_{i}$ and disease $d_{j}$, respectively. $U\in R^{m\times r}$ and $V\in R^{n\times r}$ are potential vectors for all lncRNAs and diseases. Then, the probabilities *P* of all lncRNAs and diseases were modeled by the following logistic function:

(5) $$P=\frac{\exp\left(UV^{T}\right)}{1+\exp\left(UV^{T}\right)}$$

What needs to be emphasized is the calculation of *P* depends on the lncRNA–disease association network *Y*. Based on the hypothesis that similar diseases are always associated with functionally similar lncRNAs, the interaction probability of lncRNA–disease is affected not only by the lncRNA–disease association network *Y*, but also by lncRNAs’ similarity network $S_{l}^{k}$ and diseases’ similarity network $S_{d}^{k}$. Hence, *Y* is combined with $S_{l}^{k}$ and $S_{d}^{k}$ for matrix factorization. The interaction probabilities of lncRNAs and diseases are:

(6) $$P=\frac{\exp\left(\alpha UV^{T}+\beta S_{l}^{k}UV^{T}+\gamma UV^{T}S_{d}^{k}\right)}{1+\exp\left(\alpha UV^{T}+\beta S_{l}^{k}UV^{T}+\gamma UV^{T}S_{d}^{k}\right)}$$

where α,β,γ are the corresponding weight of *Y*, $S_{l}^{k}$ and $S_{d}^{k}$. Their sum is 1, and β=γ.

Since the known lncRNA–disease associations are more important than the unknown lncRNA–disease associations, we set the weight of the known lncRNA–disease pairs to *c* (c≥1 ) and that of the unknown lncRNA–disease pairs to 1. By assuming all samples are independent, the probability $p\left(Y|U,V\right)$ can be calculated by:

(7) $$p\left(Y|U,V\right)=\prod_{i=1}^{m}\prod_{j=1}^{n}P_{ij}^{cY_{ij}}{\left(1-P_{ij}\right)}^{1-Y_{ij}}$$

where $P_{ij}$ is the interaction probability between lncRNA $l_{i}$ and disease $d_{j}$. Setting the zero-mean spherical Gaussian prior in lncRNAs’ and diseases’ potential vectors is done as follows:

(8) $$p\left(U|\sigma_{l}^{2}\right)=\prod_{i=1}^{m}N\left(u_{i}|0,\sigma_{l}^{2}I\right),p\left(V|\sigma_{d}^{2}\right)=\prod_{j=1}^{n}N\left(v_{j}|0,\sigma_{d}^{2}I\right)$$

where $\sigma_{l}^{2}$ and $\sigma_{d}^{2}$ are the parameters that control the variance of the Gaussian distribution and *I* represents the identity matrix. According to Bayesian inference:

(9) $$p\left(U,V|Y,\sigma_{l}^{2},\sigma_{d}^{2}\right)\propto p\left(Y|U,V\right)p\left(U|\sigma_{l}^{2}\right)p\left(V|\sigma_{d}^{2}\right)$$

Then, learn the model parameters *U* and *V* by maximizing the logarithm of the posterior distribution. The objective function *L* is:

(10) $$\begin{array}{c} L=\max_{U,V}\sum_{i,j}\left(cY⊙\left(\alpha UV^{T}+\beta S_{l}^{k}UV^{T}+\gamma UV^{T}S_{d}^{k}\right)-\left(1+cY-Y\right)⊙\ln\left[1+\exp\left(\alpha UV^{T}\right.\right.\right.\\ \;+\beta S_{l}^{k}UV^{T}+\gamma UV^{T}S_{d}^{k})])-\frac{\lambda_{u}}{2}{∥U∥}_{F}^{2}-\frac{\lambda_{v}}{2}{∥V∥}_{F}^{2} \end{array}$$

where $\lambda_{u}=\frac{1}{\sigma_{l}^{2}}$, $\lambda_{\nu }=\frac{1}{\sigma_{d}^{2}}$, $\lambda_{u}$, and $\lambda_{\nu }$ are regularization coefficient of *U* and *V*, ${∥\cdot ∥}_{F}^{2}$ is the Frobenius norm, and ⊙ is the Hadamard product. Starting from the above objective function, the gradient descent algorithm was used to solve *U* and *V*, and the gradient variables of *U* and *V* are as follows:

(11) $$\frac{\partial L}{\partial U}=c\left(\alpha I+\beta {\left(S_{l}^{k}\right)}^{T}\right)YV+\gamma \left(cY-Q\right){\left(S_{d}^{k}\right)}^{T}V-\left(\alpha I+\beta {\left(S_{l}^{k}\right)}^{T}\right)QV-\lambda_{u}U$$

(12) $$\frac{\partial L}{\partial V}=c\left(\alpha I+\gamma {\left(S_{d}^{k}\right)}^{T}\right)Y^{T}U+\beta \left(cY^{T}-Q^{T}\right)S_{l}^{k}U-\left(\alpha I+\gamma {\left(S_{d}^{k}\right)}^{T}\right)Q^{T}U-\lambda_{v}V$$

where $Q=\left(1+cY-Y\right)⊙\frac{1}{\exp\left(-\left(\alpha UV^{T}+\beta S_{l}^{k}UV^{T}+\gamma UV^{T}S_{d}^{k}\right)\right)+1}$, $Q^{T}$ is the transposed matrix of *Q*. This work uses the AdaGrad algorithm [38] to accelerate the convergence of *U* and *V*.

Based on the matrices *U* and *V*, the interaction probabilities of any unknown lncRNA–disease pairs can be calculated by Formula (6). Due to the uncertainty of lncRNA $l_{i}\in L^{-}$ and disease $d_{j}\in D^{-}$, their potential vectors $u_{i}$ and $v_{j}$ obtained by gradient descent cannot accurately describe their characteristics, so k-nearest neighbor sets $N^{+}\left(l_{i}\right)$ and $N^{+}\left(d_{j}\right)$ of $l_{i}$ and $d_{j}$ were constructed (k was empirically set to 5). Then, replace potential vector $u_{i}$ and $v_{j}$ with the linear combination of the k-nearest neighbors [25,26]. The modified interaction probability is:

(13) $${\hat{p}}_{ij}=\frac{\exp\left({\hat{u}}_{i}{\hat{v}}_{j}^{T}\;\;\right)}{1+\exp\left({\hat{u}}_{i}{\hat{v}}_{j}^{T}\right)}$$

where:

(14) $${\hat{u}}_{i}=\left\{\begin{array}{c} \;\;\;\;\;\;\;\;\;\;\;\;\;\;\;\;\;\;\;\;\;\;u_{i},\;l_{i}\in L^{+}\\ \frac{1}{\sum_{u\in N^{+}\left(l_{i}\right)}S_{iu}^{l}}\sum_{\;u\in N^{+}\left(l_{i}\right)}S_{iu}^{l}u_{u},\;l_{i}\in L^{-} \end{array}\right.$$

(15) $${\hat{v}}_{j}=\left\{\begin{array}{c} \;\;\;\;\;\;\;\;\;\;\;\;\;\;\;\;\;\;\;\;\;\;v_{j},\;d_{j}\in D^{+}\\ \frac{1}{\sum_{v\in N^{+}\left(d_{j}\right)}S_{jv}^{d}}\sum_{v\in N^{+}\left(d_{j}\right)}S_{jv}^{d}u_{v},\;d_{j}\in D^{-} \end{array}\right.$$

$S_{iu}^{l}$ denotes the similarity between unknown lncRNA $l_{i}$ and known lncRNA $l_{u}$, and $u_{u}$ denotes the latent variable of $l_{u}$.

The selection of model parameter $r,\alpha ,\beta ,\gamma ,\lambda_{u},\lambda_{v}$ can affect the performance of the model somehow. It is difficult to ensure the best performance of the model by using empirical parameter values. In order to improve the performance of the model, the Bayesian optimization algorithm was adopted to optimize the setting of parameter values in this work.

### 3.3. Bayesian Optimization

**Figure 2:**
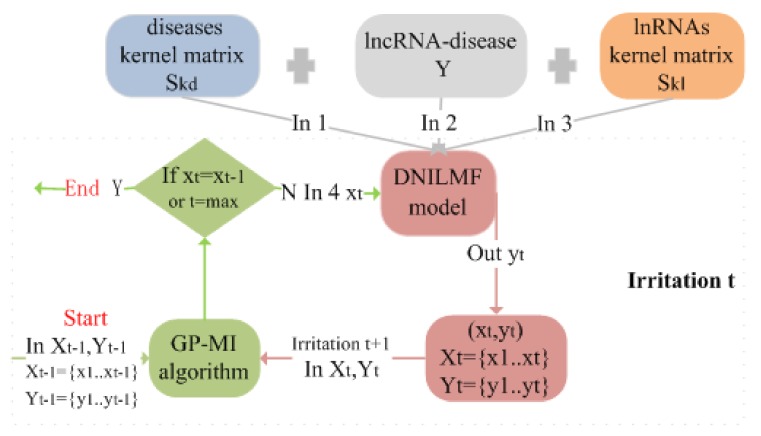
The optimization process of GP-MI for the DNILMF model parameters. At irritation t: **Step 1**: get $x_{t}$ according to the previous query $\chi_{t-1}$ and observations $Y_{t-1}$; **Step 2**: if $x_{t}$=$x_{t-1}$ or t is equal to the max value, exit the program; if not, put $x_{t}$, the disease kernel matrix, lncRNA–disease association matrix, and lncRNA kernel matrix into the DNILMF model, and we can get output $x_{t}$. Then, take $x_{t}$ and $y_{t}$ as the start of the next irritation.

The Gaussian process mutual information algorithm (GP-MI) was used to optimize the setting of the parameter values. The optimization process of GP-MI for the DNILMF model parameters is shown in Figure 2.

(1) Bayesian optimization

For function f:χ→R, *f* is an unknown function to be optimized, and $\chi \subset R^{n}\left(n\in N\right)$, a tight convex set. In this paper, the DNRLMF model is *f*, and $R^{n}$ is the parameter search space. The purpose of Bayesian optimization is to find the optimal solution for *f* through continuous queries x ($x_{1},x_{2},\ldots \in \chi$). At iteration t, the new query $x_{t}$ is selected from χ according to the previous query $\chi_{t-1}=\left\{x_{1},x_{2},\ldots x_{t-1}\right\}$ and observations $Y_{t-1}=\left\{y_{1},y_{2},\ldots ,y_{t-1}\right\}$. The relationship between $y_{t}$ and $x_{t}$ is $y_{t}=f\left(x_{t}\right)+\epsilon_{t}$, where $\epsilon_{t}$ is the noise variable, $\epsilon_{t}∼N\left(0,\sigma^{2}\right)$.

(2) Gaussian process

Suppose the function *f* follows Gaussian process $GP\left(m,k\right)$ [30], where m:χ→R is a mean function and k:χ×χ→R is a kernel function. Let the mean function be zero, that is m:χ→0, the kernel function is a square exponential kernel.

According to the previous t−1 times queries $\chi_{t-1}$ and observations $Y_{t-1}$, the posterior distribution at iteration *t* is a Gaussian process with expectation as $\mu_{t}\left(x\right)$ and variance as $\sigma_{t}^{2}\left(x\right)$ by Bayesian inference.

(3) GP-MI algorithm

The most critical aspect of the GP-MI algorithm is the choice of the next query $x_{t}\in \chi$ using $\mu_{t}\left(x\right)$ and variance $\sigma_{t}^{2}\left(x\right)$.

(16) $$x_{t}=\underset{x\in \chi }{argmax}\mu_{t}\left(x\right)+\phi_{t}\left(x\right)$$

where $\phi_{t}:\chi \to R$ is the increment function of $\sigma_{t}^{2}\left(x\right)$:

(17) $$\phi_{t}\left(x\right)=\sqrt{\log\frac{2}{\delta }}\left(\sqrt{\sigma_{t}^{2}\left(x\right)+{\hat{\gamma }}_{t-1}}-\sqrt{{\hat{\gamma }}_{t-1}}\right)$$

${\hat{\gamma }}_{t-1}\leftarrow {\hat{\gamma }}_{t-2}+\sigma_{t-1}^{2}\left(x_{t-1}\right)$; δ>0 is a hyperparameter; and the iteration ending condition is $x_{t+1}=x_{t}$. The pseudocode of the GP-MI is shown in Algorithm 1:

**Algorithm 1** GP-MI.

${\hat{\gamma }}_{0}\leftarrow 0$


**for** t=1,2,… **do**

 Compute $\mu_{t}$ and $\sigma_{t}^{2}$ by $\chi_{t}=\left\{x_{1}..x_{t-1}\right\}$ and $Y_{t}=\left(y_{1}..y_{t-1}\right)$ *// Bayesian inference*
 $\phi_{t}\left(x\right)\leftarrow \sqrt{\log\frac{2}{\delta }}\left(\sqrt{\sigma_{t}^{2}\left(x\right)+{\hat{\gamma }}_{t-1}}-\sqrt{{\hat{\gamma }}_{t-1}}\right)$ *// Definition of $\phi_{t}\left(x\right)$ for all x∈χ*

 $x_{t}\leftarrow \underset{x\in \chi }{argmax}\mu_{t}\left(x\right)+\phi_{t}\left(x\right)$ *// Selection of the next query location*
 ${\hat{\gamma }}_{t}\leftarrow {\hat{\gamma }}_{t-1}+\sigma_{t}^{2}\left(x_{t}\right)$ *// Update ${\hat{\gamma }}_{t}$*
 get $y_{t}$ by the DNILMF model and $x_{t}$ *// Query $\left(x_{t},y_{t}\right)$*


**end for**



## 4. Experimental Results

### 4.1. Evaluation of Prediction Performance

In this paper, the prediction performance of the detection model was verified by 10-fold cross-validation (CV). AUC and the area under precision-recall (PR) curve (AUPR) were used as the performance evaluation indexes of the model. AUC is an important index to evaluate the classification model. If AUC = 1, the model has perfect performance; if AUC = 0.5, this means random performance. The higher the values of AUC and AUPR, the better the prediction performance.

During the 10-fold CV process, lncRNA–disease pairs (including known pairs and unknown pairs) were randomly divided into ten groups with almost the same data size by setting random seeds. Each time, one of the ten groups was used as the test data, and the values of the test data in the adjacency matrix *Y* were set to zero. The resulting matrix was the training data $Y_{train}$. In each iteration of 10-fold CV, firstly, calculate the kernel matrix and the GIP kernel matrix of lncRNAs and diseases. Secondly, fuse the kernel matrices of lncRNAs and diseases to get two composite kernel matrices. Then, take the fused kernel matrices and $Y_{train}$ as the model input and update the value of the potential vectors *U*, *V* through gradient descent until the optimal value of the model is achieved. Finally, the AUC and AUPR values were obtained by using the trained model to predict and evaluate the test data. After ten iterations, the AUC values of 10 test sets were obtained, and their mean value was taken as the AUC value of one time 10-fold CV. Under 10-fold CV, the AUC value of the model reached 0.9202, and the AUPR value reached 0.5610.

### 4.2. Comparison with Other Methods

**Figure 3:**
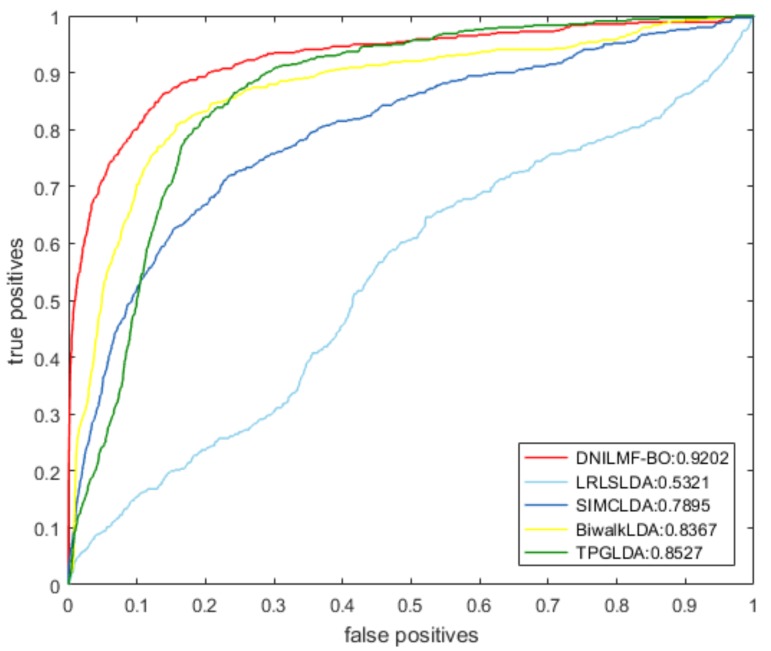
ROC curve of the five models.

**Figure 4:**
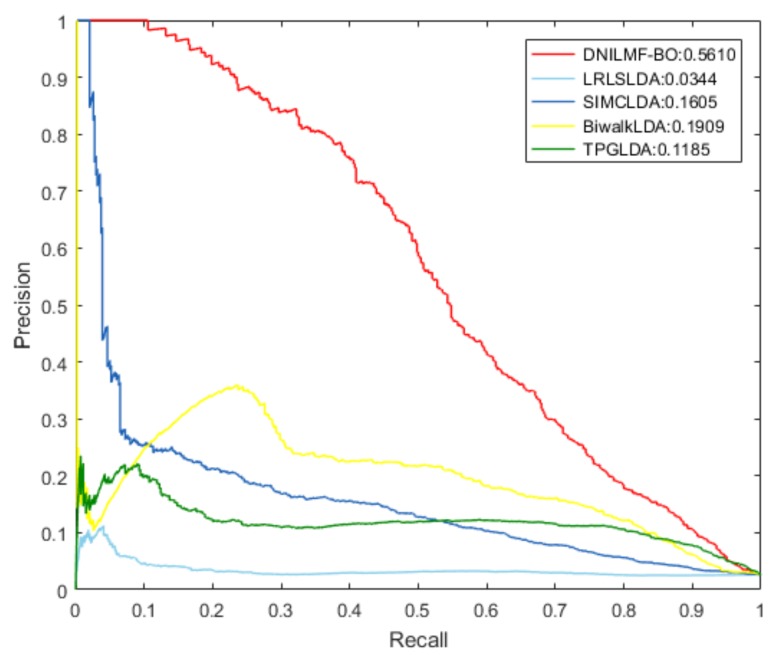
PR curve of the five models.

To further evaluate the performance of our model, we compared it with LRLSLDA, BiwalkLDA, SIMCLDA, and TPGLDA under 10-fold CV. The prediction result of the five models using the same dataset is shown in Table 1. The result showed that both AUC and AUPR values of DNILMF-LDA were the highest among five models, indicating that the performance of our model was better than the others. Figure 3 and Figure 4 respectively show the receiver operating characteristic (ROC) curve and precision-recall (PR) curve of the five models.

### 4.3. Parameter Analysis

For DNILMF-LDA, the dimension *r* of shared potential space was from 50–100 with a step length of 10 [31]. The coefficient of the potential matrix product ranged from 0–1 with a step length of 0.1, $\beta =\gamma =\left(1-\alpha \right)/2$; the regularization coefficients $\lambda_{u}$ and $\lambda_{v}$ for potential variables of lncRNAs and diseases ranged from 1–10, with a step size of one [25]. The number of neighbors to construct the neighbor set of unknown lncRNAs and diseases was set to five. The weight of known interaction pair was set to five. According to the results of the literature [31], when $\delta =10^{-100}$, the Bayesian optimization was very close to the prediction accuracy of the grid search, but the calculation time decreased by 8.94-times on average. Therefore, we set the value of δ and the noise variance of the Gaussian process kernel function $\sigma^{2}$ to $10^{-100}$ and 0.1, respectively. In summary, $r=\left\{50,100\right\}$, α={0.1,1}, $\lambda_{u}=\left\{1,10\right\}$, $\lambda_{v}=\left\{1,10\right\}$, K=5, c=5, $\delta =10^{-100}$, and $\sigma^{2}=0.1$.

**Figure 5:**
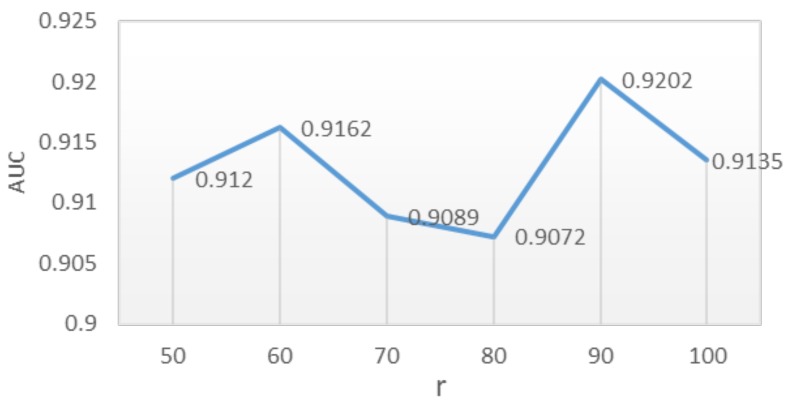
Influence of *r* on AUC value when α=0.1, β=γ=0.45, $\lambda_{u}=1$, $\lambda_{v}=1$.

The parameter optimization results of the DNILMF model by the GP-MI algorithm showed that the prediction performance of the model was good when the model parameters *r* took any value in the range of $\left\{50,100\right\}$, α=0.1, β=γ=0.45, $\lambda_{u}=1$, $\lambda_{v}=1$. When r=90, the AUC value of the model reached its highest at 0.9202. The AUC value is shown in Figure 5 when *r* took different values. The weight of β and γ was greater than that of *c*, which indicated the importance of the lncRNAs’ and diseases’ similarity network and also indicated the effectiveness of adding the lncRNA–disease associations network and similarity networks into the model.

### 4.4. Case Studies on Breast, Lung, and Colon Cancer

We further evaluated the role of the DNILMF-LDA model in predicting lncRNA–disease associations by studying three common and typical cancers: breast cancer, lung cancer, and colon cancer. The top ten candidate lncRNAs calculated by DNILMF-LDA for three cancers and their evidence are listed in Table 2, Table 3 and Table 4. The verification of the prediction results was supported by the lncRNADisease and lnc2Cancer databases [14,15].

Lung cancer is one of the most common and deadly cancers in the world. Among the top 10 candidate lncRNAs calculated by DNILMF-LDA, seven lncRNAs were experimentally verified to be associated with lung cancer. For example, the lncRNA-CDKN2B-AS1 promotes NSCLC cell proliferation and inhibits apoptosis by suppressing KLF2 and P21 expression [39]. In addition, a recent study has shown that upregulated lncRNA-UCA1 plays an important role in the development of lung cancer, and it has great application prospects in clinical diagnosis [40].

Colon cancer is the third most common cancer and the second leading cause of cancer death in men and women [41]. Of the top 10 candidate lncRNAs calculated by DNILMF-LDA, eight lncRNAs were experimentally demonstrated to be associated with colon cancer. Studies have shown that inhibiting the expression of lncRNA-TUG 1 can significantly inhibit the migration ability of colon cancer cells, and the overexpression of TUG 1 may promote the proliferation and migration of colon cancer cells [42].

Breast cancer is the most common cancer in women and the most common cancer in the world. Among the top ten candidate lncRNAs calculated by DNILMF-LDA, seven lncRNAs were experimentally demonstrated to be associated with breast cancer. Studies have shown that upregulated lncRNA-CCAT 1, second in our list of breast cancer, participates in various cellular processes related to cancer occurrence [43].

These case studies reconfirmed the potential of DNILMF-LDA in identifying potential lncRNA–disease associations.

## 5. Discussion

Studies have shown that lncRNAs play an essential role in biological processes and in the diagnosis, prevention, and treatment of complex diseases. It has become an extraordinary method to combine multiple different similarity matrices in the computational model, and using matrix factorization to predict the potential lncRNA–disease associations is also a hot topic. In this paper, the dual-network integrated logistic matrix factorization model was used to predict the potential lncRNA–disease associations, and the GP-MI algorithm of Bayesian optimization was applied for parameter optimization to ensure the optimal performance of the model.

The main advantages of DNILMF-LDA are: (1) Logistic matrix factorization, especially suitable for binary variables and sparsity problems, was used to model the associations probability of each lncRNA–disease pair. (2) The GIP kernel matrix and similarity matrix of lncRNAs and diseases were obtained, and the nonlinear fusion method was adopted in the process of similarity kernel fusion to reduce the difference between similarity matrices. (3) lncRNAs’ and diseases’ similarity networks were introduced in the model. In this paper, 10-fold CV was used to evaluate the prediction performance of our model. The results showed that compared with the LRLSLDA, BiwalkLDA, SIMCLDA, and TPGLDA models, the AUC value of DNILMF-LDA was higher and the prediction performance of DNILMF-LDA better. In addition, case studies of lung cancer, colon cancer, and breast cancer also suggested that DNILMF-LDA was a better computational method to predict the potential lncRNA–disease associations.

Although DNILMF-LDA has obtained reliable experimental results, there are still some biases. For example, the known experimentally-verified lncRNA–disease associations are still limited, and the predictive performance of DNILMF-LDA will be improved by a more comprehensive dataset.

## 6. Conclusions

In this paper, our major contributions were as follows: First, logistic matrix factorization was used to model the interaction probability of each lncRNA–disease pair. Second, lncRNA and disease similarity networks were introduced into the model. Third, the imbalance between known and unknown interaction pairs was balanced by giving higher weights to known interactions in the model. Fourth, the method of neighborhood information was used to deal with the problems of new lncRNAs and diseases in the process of prediction. Fifth, multiple source similarity fusion was used to improve the prediction accuracy. We obtained the Gaussian kernel matrix and similarity kernel matrix of lncRNAs and diseases, adopted nonlinear fusion to weaken the differences between similar matrices, and extracted the most important information from different similarity data. Sixth, the GP-MI algorithm in Bayesian optimization was adopted in this paper for parameter optimization.

In the future, we expect to acquire new multi-source datasets and explore better kernel fusion methods. Then, we can improve the prediction performance by fully exploiting multi-source data and advanced fusion technology.

## Figures and Tables

**Table 1:** AUC and area under precision-recall (PR) curve (AUPR) values of the five models.

Method	AUC	AUPR
LRLSLDA	0.5321	0.0344
SIMCLDA	0.7895	0.1605
BiwalkLDA	0.8367	0.1909
TPGLDA	0.8527	0.1185
DNILMF-LDA	0.9202	0.5610

**Table 2:** The top ten lncRNA candidates for lung cancer.

Top	lncRNA	Evidence	Description
1	CCAT2	26729200	lncRNADisease
2	CDKN2B-AS1	26729200	lncRNADisease
3	PVT1	28731781	lnc2Cancer
4	UCA1	29731641	lnc2Cancer
5	CCAT1	27212446	lncRNADisease
6	SPRY4-IT1	26302345	lncRNADisease
7	GAS5	26634743	lncRNADisease
8	HULC	unconfirmed	unconfirmed
9	SRA1	unconfirmed	unconfirmed
10	XIST	unconfirmed	unconfirmed

**Table 3:** The top ten lncRNA candidates for colon cancer.

Top	lncRNA	Evidence	Description
1	SPRY4-IT1	28099409	lnc2Cancer
2	HOTTIP	26617875	lnc2Cancer
3	GHET1	27931286	lnc2Cancer
4	MINA	unconfirmed	unconfirmed
5	HIF1A-AS2	29278853	lnc2Cancer
6	ADAMTS9-AS2	27596298	lnc2Cancer
7	TUG1	28302487	lnc2Cancer
8	LINC00152	29180678	lnc2Cancer
9	PANDAR	28176943	lnc2Cancer
10	BC040587	unconfirmed	unconfirmed

**Table 4:** The top ten lncRNA candidates for breast cancer.

Top	lncRNA	Evidence	Description
1	MNX1-AS1	unconfirmed	unconfirmed
2	CCAT1	26464701	lnc2Cancer
3	TUSC7	23558749	lnc2Cancer
4	BANCR	29565494	lnc2Cancer
5	DNM3OS	unconfirmed	unconfirmed
6	TUG1	27791993	lncRNADisease
7	RPL34-AS1	unconfirmed	lncRNADisease
8	MINA	25586347	lnc2Cancer
9	GHET1	29843220	lnc2Cancer
10	PTENP1	29085464	lnc2Cancer
